# Morphological plasticity in *Myxobolus* Bütschli, 1882: a taxonomic dilemma case and renaming of a parasite species of the common carp

**DOI:** 10.1186/s13071-018-2943-0

**Published:** 2018-07-09

**Authors:** Qingxiang Guo, Mingjun Huang, Yang Liu, Xiuping Zhang, Zemao Gu

**Affiliations:** 1 Department of Aquatic Animal Medicine, College of Fisheries, Huazhong Agricultural University, Wuhan, Hubei Province 430070 People’s Republic of China; 2 Hubei Engineering Technology Research Center for Aquatic Animal Diseases Control and Prevention, Wuhan, Hubei Province 430070 People’s Republic of China

**Keywords:** Myxozoan, *Myxobolus pseudoacinosus*, Morphotype, SSU rDNA, Taxonomic dilemma, Tissue tropism

## Abstract

**Background:**

Myxozoans are a group of cnidarian parasites, the present taxonomy of which favors a more comprehensive characterization strategy combining spore morphology, biological traits (host/organ specificity, tissue tropism), and DNA data over the classical morphology-based taxonomy. However, a systematist might again run into a taxonomic dilemma if more than two of the following exceptional cases were encountered at the same time: extensive intraspecific polymorphism, interspecific morphological similarity, identical interspecific biological traits and blurred small-subunit (SSU) rDNA-based species boundaries. In the present study, spores of a species of *Myxobolus* Bütschli, 1882 with two morphotypes (wide type and narrow type) were collected from the gills of common carp *Cyprinus carpio* Linnaeus. Confusingly, the wide type was found to be identical to *Myxobolus paratoyamai* Kato, Kasai, Tomochi, Li & Sato, 2017 in spore morphology and SSU rDNA sequence, which confidently suggested their conspecificity; while the narrow type, was highly similar to *Myxobolus toyamai* Kudo, 1917 based on spore morphology and SSU rDNA sequence and thus could not be easily classified. This discordance between wide type and narrow type has caused a taxonomic dilemma. To address this problem, a hypothesis about the conspecificity of the narrow type and *M. toyamai* was addressed.

**Results:**

It was found that if the narrow type is conspecific with *M. toyamai*, it would be paradoxical for the SSU rDNA sequence of the narrow type to be more similar to *M. paratoyamai* (99.3%), *Myxobolus acinosus* Nie & Li, 1973 (98.6%) and *Myxobolus longisporus* Nie & Li, 1992 (98.7%) than to *M. toyamai* (97.6%). According to the results of the above what-if analysis, the narrow type and *M. toyamai* were considered to be different species. All in all, the present dual-morphotype species is estimated to be conspecific with *M. paratoyamai* Kato, Kasai, Tomochi, Li & Sato, 2017. Considering that this species name was preoccupied by *Myxobolus paratoyamai* Nie & Li, 1992, the replacement name *Myxobolus pseudoacinosus* nom. nov. is proposed.

**Conclusions:**

This work addresses the taxonomic dilemma in polymorphic myxozoans and demonstrates that *M*. *pseudoacinosus* is a distinct species with two morphotypes. The present study may serve as a baseline for future studies that encounter similar classification complexities.

**Electronic supplementary material:**

The online version of this article (10.1186/s13071-018-2943-0) contains supplementary material, which is available to authorized users.

## Background

Myxozoans are widespread cnidarian parasites with over 2400 species recorded [1]. Most of them were initially described using traditional taxonomy methods based solely on spore morphology [2]. Later on, myxozoan systematists resorted to also using host preference/tissue specificity to aid in resolving taxonomic problems, with the awareness that most myxozoans tend to be oioxenic and have preference for certain developmental sites [3–5]. Moreover, the molecular methods involving genetic markers have boomed in the past decade and provided a more comprehensive solution to resolving morphologically indistinguishable species by taking advantage of their fidelity and quantifiability [4, 6]. Taken together, the above three criteria (spore morphology, biological traits and DNA data) have constituted a well-accepted framework for myxozoan classification [7].

However, there are exceptional cases that involve extensive intraspecific polymorphism, interspecific morphological similarity, identical interspecific biological traits, and blurred SSU rDNA-based species boundaries [8–11]. In these situations, if more than two of above exceptional cases are encountered at the same time, a systematist might again run into what we call a “taxonomic dilemma”. Unfortunately (fortunately?), this is the exact situation that we are facing.

In the present study, a species of *Myxobolus* with two morphotypes (wide type and narrow type) was collected from the gills of the common carp *Cyprinus carpio* Linnaeus. Paradoxically, the wide-type spores were found to be identical to *Myxobolus paratoyamai* Kato, Kasai, Tomochi, Li & Sato, 2017 based on spore morphology and SSU rDNA sequence, while the narrow-type spores were highly similar to *Myxobolus toyamai* Kudo, 1917 both in spore morphology and SSU rDNA sequence. Three of the four above mentioned exceptional cases (intraspecific polymorphism, interspecific morphological similarity and blurred SSU rDNA-based species boundaries) were herein encountered and created this taxonomic dilemma. In order to resolve the current uncertainties in species identification, the present material was characterized considering both morphology and molecular biology data and was subsequently determined to be conspecific with *M. paratoyamai* [12], with the additional trait of having two morphotypes.

## Methods

### Fish sampling and morphological analysis

Common carp *C. carpio* were sampled from the Baishazhou Fish Market, Wuhan, China in January 2013 (*n* = 34; total length 16–24 cm) and April 2015 (*n* = 4; total length 23–28 cm). Fish were sent to the laboratory and kept in a relaying tank prior to being euthanized with an overdose of MS-222 (Sigma-Aldrich, Co., Ltd., St. Louis, MO., USA). Parasitological examinations were then conducted on the specimens and fresh myxospores were visualized and photographed under an Olympus BX53 light microscope using Nomarski differential interference contrast and, equipped with an Olympus DP73 digital camera (Olympus, Hamburg, Germany). Myxozoan identification and morphological analysis were performed following the formerly developed guidelines [13] based on morphometric measurements of 40 fresh mature spores. All measurements are shown in micrometres (μm) as the range, followed by the mean ± SD in parentheses.

### Histopathological examination and ultrastructure

Fish gills containing plasmodia were fixed with Bouin’s solution, gradient-dehydrated, embedded in paraffin wax and sectioned at 4 μm, and stained with haematoxylin and eosin. For transmission electron microscopy, tissues containing the plasmodia were excised and the following fixation, dehydration, embedment and staining steps were conducted according to the protocol of Liu et al. [14]. Double-stained sections were visualized and photographed using a 200 kV transmission electron microscope (Tecnai G20 TWIN, FEI company, OR, USA).

### DNA extraction, amplification and sequencing

Ethanol-preserved plasmodia were used for genomic DNA extraction according to the protocols recommended by the manufacturer of the TIANamp Genomic DNA Kit (Beijing Tiangen Biotech Co. Ltd., China). The SSU rRNA gene was amplified with universal eukaryotic primer pairs 18e [15] and 18r [16], for spores collected in 2015 (narrow type), or MyxospecF [17] and SphR [18], for spores collected in 2013 (wide type). The next step of PCR, purification, cloning and transformation were performed following the procedure of Zhao et al. [19]. DNA sequences were then defined bidirectionally by Sanger sequencing on an ABI PRISM® 3730 sequencer (Applied Biosystems Inc., Foster, USA), with the resulting contiguous sequences assembled by the Lasergene package v 5. 05 (DNASTAR, Madison, Wisconsin) and corrected with BioEdit 7.2.5 [20] based on the original sequence chromatograms. Contiguous sequences were then submitted to an NCBI BLASTn search for comparison with other myxozoan sequences.

### Phylogenetic and distance analysis

The newly acquired SSU rDNA sequences of *M. pseudoacinosus* and 79 other myxozoan sequences retrieved from GenBank were used for phylogenetic analysis. *Myxidium streisingeri* Whipps, Murray & Kent, 2015 (GenBank: KM001688) was selected as the outgroup. Alignments were performed with MAFFT v7.305b [21] using default parameters. Gblocks [22] was used to remove unreliably aligned regions (with the following parameters: -t = d, -b1 = 41, -b2 = 41, -b3 = 10, -b4 = 2, -b5 = a), resulting in a final data set of 81 taxa and 1713 positions. Substitution saturation was evaluated with DAMBE 5 [23]. Phylogenetic analyses were conducted with RAxML v8.2.9 (maximum likelihood analysis; [24]) and Mr. Bayes 3. 2.6 (Bayesian inference; [25]), employing the GTR+I+G model calculated by Jmodeltest2 [26] using the Akaike Information Criterion (AIC). For maximum likelihood analysis, 1000 bootstrap pseudoreplicates were performed. For Bayesian inference, 3,000,000 generations of Markov chain Monte Carlo simulations were run. For every 100 generations, tree-sampling was conducted and the initial 7500 trees were taken out as ‘burn-in’. Chain convergence was examined with Tracer v1.6 [27]. Distance estimation was carried out between nine closely related sequences, using the p-distance model of substitution in FastME 2.0 [28]. The alignments were trimmed so as to eliminate nucleotide positions with gaps, resulting in a final data set of 796 characters. Visualization of the genetic distance matrix was generated using a custom Python script.

## Results

**Fig. 1:**
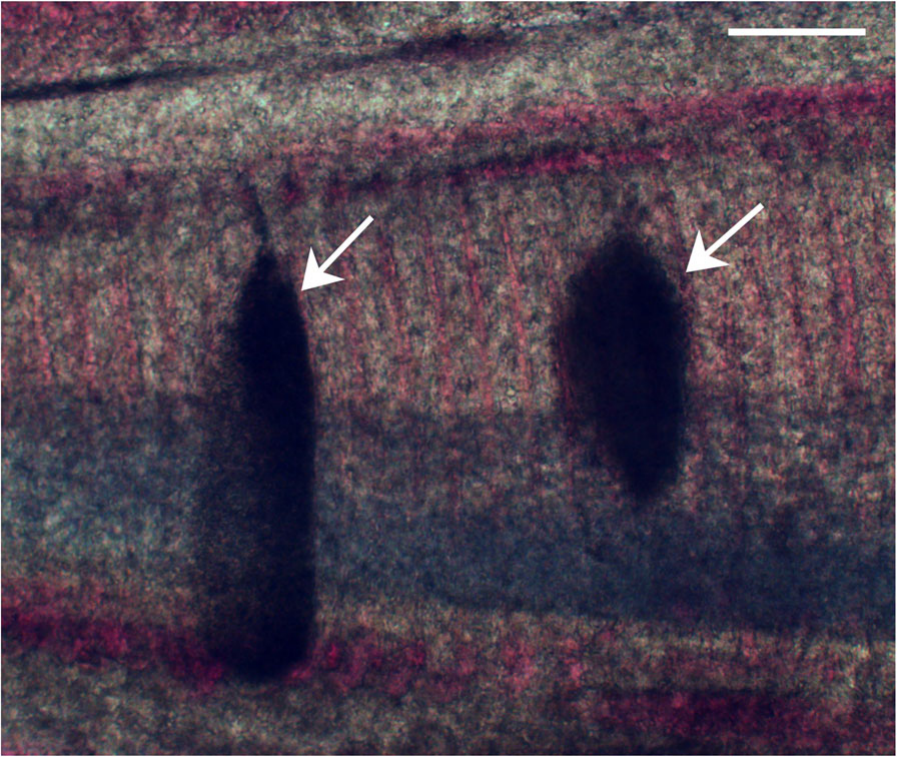
Gills of the common carp *Cyprinus carpio* heavily infected with *Myxobolus pseudoacinosus*. Note the plasmodia (arrows). *Scale-bar*: 300 μm

Parasitological examination revealed that numerous whitish and ellipsoidal plasmodia measuring up to ~660 μm were located in the gill lamellae of infected common carp specimens (Fig. 1). After rupturing the plasmodia, mature spores with two unequal polar capsules, one of which is extremely stunted, were observed, thus defining classification within the genus *Myxobolus*.

**Family Myxobolidae Thélohan, 1892**

**Genus** ***Myxobolus*** **Bütschli, 1882**

***Myxobolus pseudoacinosus*** **nom. nov.**

Syn. *Myxobolus paratoyamai* Kato, Kasai, Tomochi, Li & Sato, 2017 *nec Myxobolus paratoyamai* Nie & Li, 1992

***Host*:** *Cyprinus carpio* L. (Cypriniformes: Cyprinidae), common carp.

***Locality*:** Baishazhou Fish Market (30°28'47"N, 114°21'20"E), Wuhan, China.

***Site in host*:** Gills.

***Voucher material*:** Giemsa-stained slides with mature spores of *M. pseudoacinosus* were deposited in the National Zoological Museum of China, Institute of Zoology, Chinese Academy of Sciences (IZCAS) under the accession numbers MTR20150428 (narrow type) and MTR20130121 (wide type).

***Prevalence*:** 25% (1/4) in 2015 specimen; 2.9% (1/34) in 2013 specimen.

***Representative DNA sequences***: GenBank accession numbers: KX586684 (narrow type); KX810019, KX810020 (wide type).

***ZooBank registration*:** To comply with the regulations set out in article 8.5 of the amended 2012 version of the *International Code of Zoological Nomenclature* (ICZN) [29], details of *Myxobolus pseudoacinosus* have been submitted to ZooBank. The Life Science Identifier (LSID) for the article is urn:lsid:zoobank.org:act:442CC572-59E4-448B-8370-FCC2ACEACB70.

***Etymology*:** The species is named after its morphological similarity to *Myxobolus acinosus* Nie & Li, 1973.

### Description

**Fig. 2:**
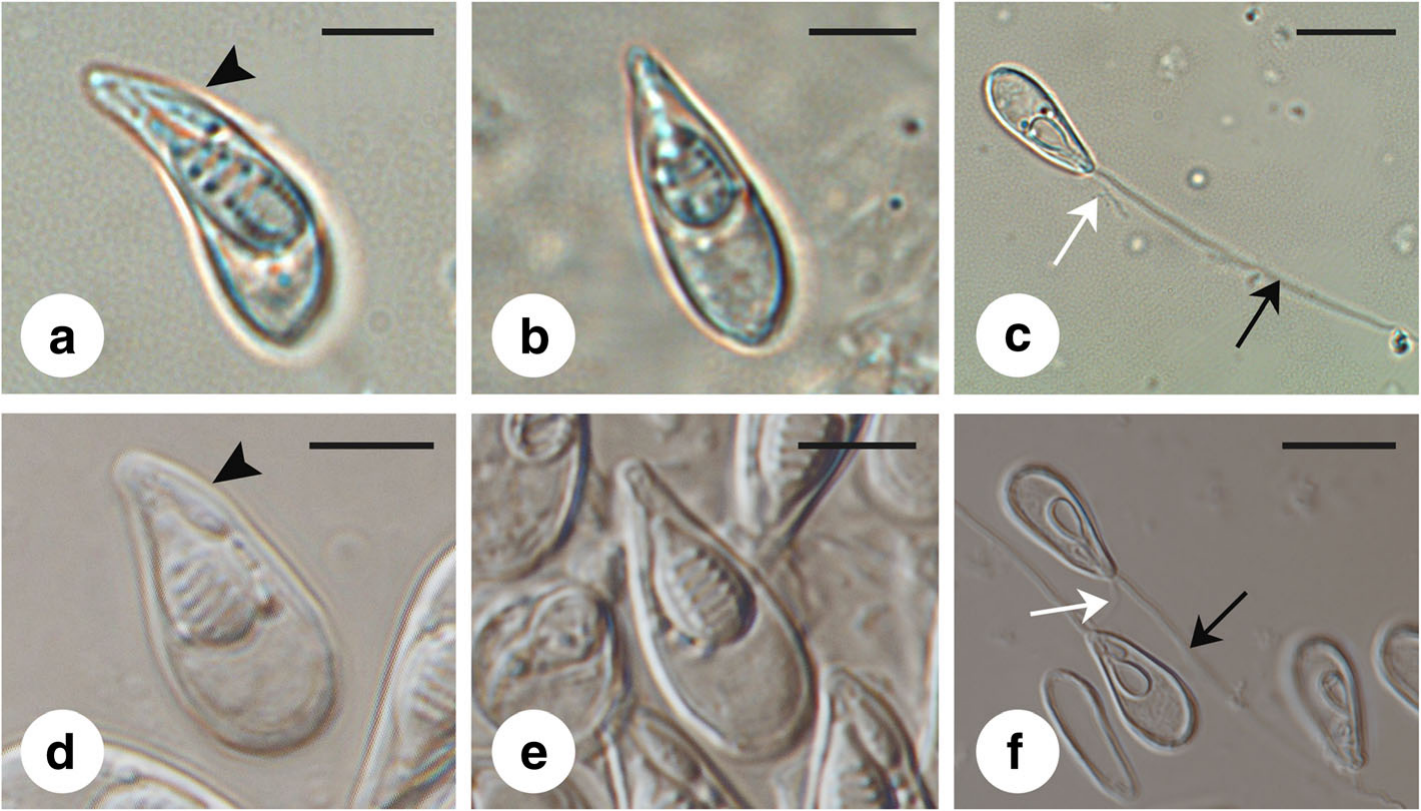
Fresh spores of *Myxobolus pseudoacinosus*. **a-c** The narrow type. **d-f** The wide type. **a, d** Spore in frontal view. Note the stunted polar capsule (arrowhead). **b, e** Spore in sutural view. **c, f** Spore with polar filaments extruded. The white and black arrows indicate the short and long polar filaments from the small polar capsule and large polar capsule, respectively. *Scale-bars*: **a**, **b**, **d**, **e**, 5 μm; **c**, **f**, 10 μm

**Fig. 3:**
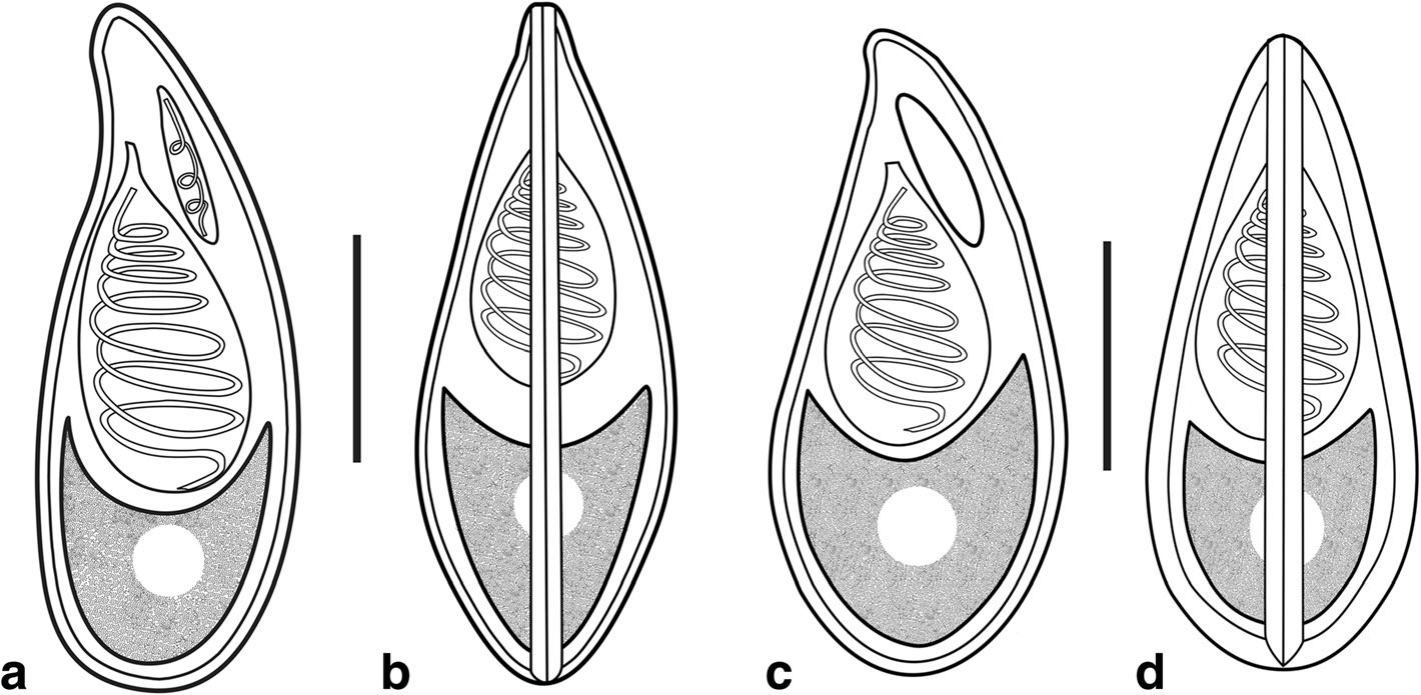
Schematic drawing of mature spores of *Myxobolus pseudoacinosus*. **a**, **b** The narrow type. **c**, **d** The wide type. **a, c** Frontal view. **b, d** Sutural view. *Scale-bars*: 5 μm

Mature spores eggplant shaped in the valvular view, protruding anteriorly, bluntly rounded posteriorly; in sutural view, spores pear-shaped, pointing and curving anteriorly. Extrusion of both short and long polar filaments obvious (Fig. 2c, f). Narrow-type spores (Figs. 2a-c, 3a, b) measuring 14.3–15.9 (15.0 ± 0.5) × 4.3–6.6 (5.5 ± 0.5) × 4.4–6.2 (5.0 ± 0.3); large polar capsule 5.7–7.8 (6.6 ± 0.5) × 2.2–3.9 (2.9 ± 0.4); small polar capsule 3.0–4.3 (3.6 ± 0.3) × 0.6–1.6 (1.0 ± 0.2). Polar filaments inside large polar capsule with 7–8 turns; polar filament of stunted polar capsule with 2–3 turns. Wide-type spores (Figs. 2 d-f, 3c, d) measuring 12.9–15.6 (14.1 ± 0.5) × 6.0–7.3 (6.5 ± 0.3) × 5.2–5.8; large polar capsule 6.0–7.3 (6.5 ± 0.3) × 3.2–4.2 (3.7 ± 0.2); small polar capsule 2.8–3.8 (3.2 ± 0.3) × 0.9–1.4 (1.2 ± 0.1).

### Remarks

**Fig. 4:**
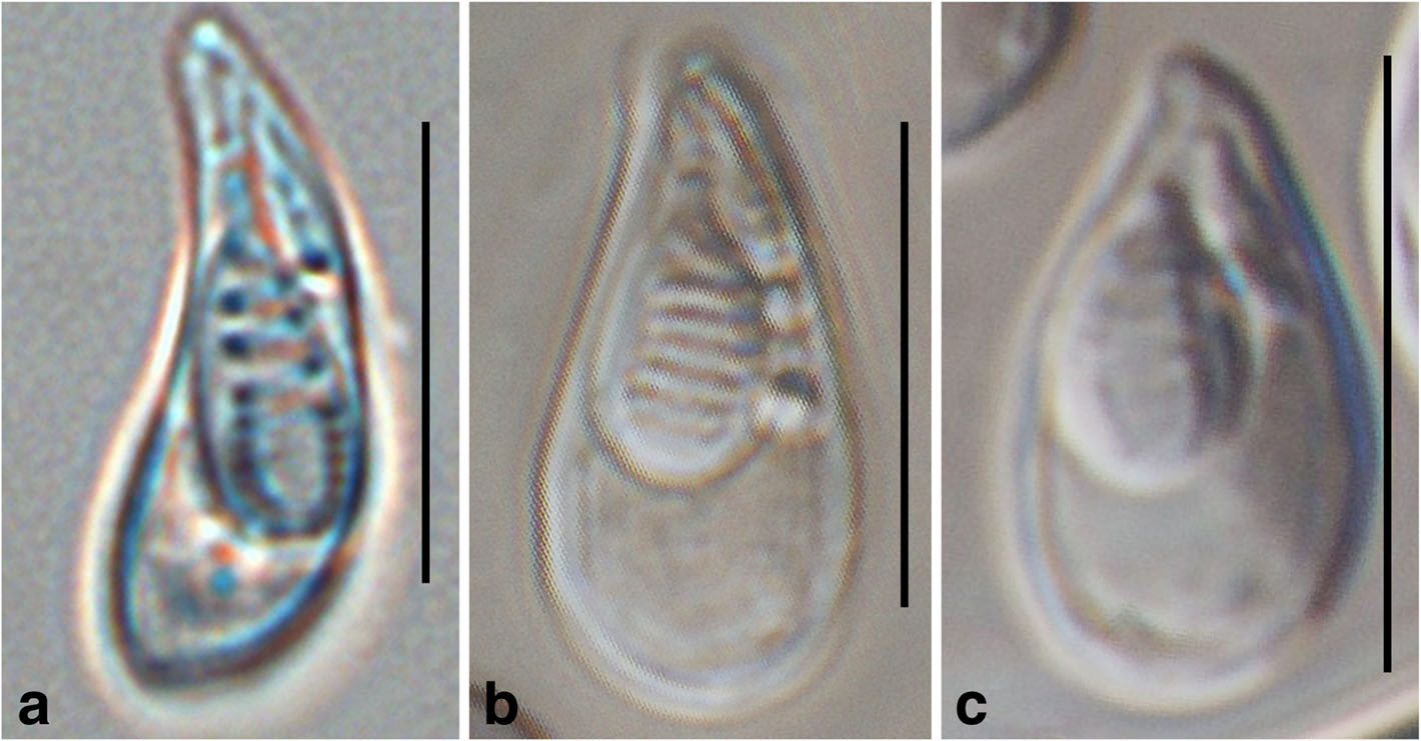
Mature spores of *Myxobolus pseudoacinosus* and *Myxobolus acinosus* Nie & Li, 1973. **a** *M. pseudoacinosus* narrow type. **b** *M. pseudoacinosus* wide type. **c** *M. acinosus* (unpublished data by Huang & Gu). *Scale-bars*: 10 μm

Although lacking molecular data, *M. paratoyamai* Nie & Li, 1992 clearly shows different morphology (shorter spore length, smaller and more posterior small polar capsule) and infection sites (nares, ureter) from the present species. *Myxobolus acinosus* Nie & Li, 1973 can be readily distinguished from both narrow type and wide type by its much shorter spore shape and shorter large polar capsule. Notably, while *M*. *toyamai* and the wide type clearly differ in spore width, it is hard to discriminate *M. paratoyamai* Kato, Kasai, Tomochi, Li & Sato, 2017 from the wide type based solely on morphology because they have highly similar morphology parameters. Similarly, *M. toyamai* and the narrow type also could not be morphologically distinguished. Details of above comparative metrical data and images of the present species and *M. acinosus* are presented in Table 1 and Fig. 4. The original images of *M. acinosus*, *M. paratoyamai* Nie & Li, 1992 and *M. toyamai* of Griffin & Goodwin, 2011 can be accessed in [2, 30, 31] respectively.

**Table 1:** Comparative measurements (in μm) shown as range (mean ± SD) of *Myxobolus pseudoacinosus* and morphologically similar *Myxobolus* spp. from *Cyprinus carpio*

Species	Country	Infection site	LS	WS	TS	LLPC	WLPC	LSPC	WSPC	NCF	Reference
Narrow type	China	Gills	14.3–15.9 (15.0 ± 0.5)	4.3–6.6 (5.5 ± 0.5)	4.4–6.2 (5.0 ± 0.3)	5.7–7.8 (6.6 ± 0.5)	2.2–3.9 (2.9 ± 0.4)	3.0–4.3 (3.6 ± 0.3)	0.6–1.6 (1.0 ± 0.2)	7–8	Present study
Wide type	China	Gills	12.9–15.6 (14.1 ± 0.5)	6.0–7.3 (6.5 ± 0.3)	5.2–5.8	6.0–7.3 (6.5 ± 0.3)	3.2–4.2 (3.7 ± 0.2)	2.8–3.8 (3.2 ± 0.3)	0.9–1.4 (1.2 ± 0.1)	6–8	Present study
*M. paratoyamai*	Japan	Gills	14.7–16.4 (15.4)	5.5–6.8 (6.3)	5.6–6.4 (6.1)	5.9–7.1 (6.5)	3.1–4.2 (3.7)	–	–	5–6	[12]
*M. paratoyamai*	China	Nares, ureter	12.5–14.2	5.5–7.0	5.0	6.2–7.4	2.2–2.5	–	–	–	[2]
*M. acinosus*	China	Gills	10.8–13.2 (12.6)	5.6–7.2 (6.4)	4.8–6.0 (5.3)	4.8–6.0 (5.3)	2.4–3.4 (2.8)	2.4–3.0	1.0–1.4	5–6	[2]
*M. acinosus*	China	Gills	10.1–11.3 (10.7 ± 0.4)	5.5–6.7 (5.9 ± 0.3)	–	4.3–5.4 (4.9 ± 0.3)	2.7–3.3 (3.0 ± 0.2)	2.6–3.3 (3.0 ± 0.3)	1.0–1.5 (1.3 ± 0.2)	7–8	Huang & Gu (unpublished)
*M. toyamai*	Japan	Gills	15.0	7.0–8.0	–	7.0–8.0	3.0–4.0	–	–	–	[38]
*M. toyamai*	China	Gills, kidneys	13.2–15.6 (14.0)	4.8–6.0 (5.5)	4.4–5.4	5.7	2.4–3.6	2.4–3.6	0.6–1.0	7–8	[2]
*M. toyamai*	USA	Gills	14.7–16.8 (16.2)	4.5–6.0 (5.6)	–	5.8–7.2 (6.4)	3.4–4.6 (4.2)	–	–	–	[31]
*M. toyamai*	Japan	Gills	13.5–15.8 (14.3)	4.5–6.3 (5.5)	–	5.0–6.8 (5.8)	2.3–4.5 (3.5)	2.7–4.5 (3.4)	0.5–1.4 (0.8)	–	[39]

*Abbreviations*: LS, spore length; WS, spore width; TS, spore thickness; LLPC, large polar capsule length; WLPC, large polar capsule width; LSPC, small polar capsule length; WSPC, small polar capsule width; NCF, number of polar filament coils; –, not available

### Histopathological examination

**Fig. 5:**
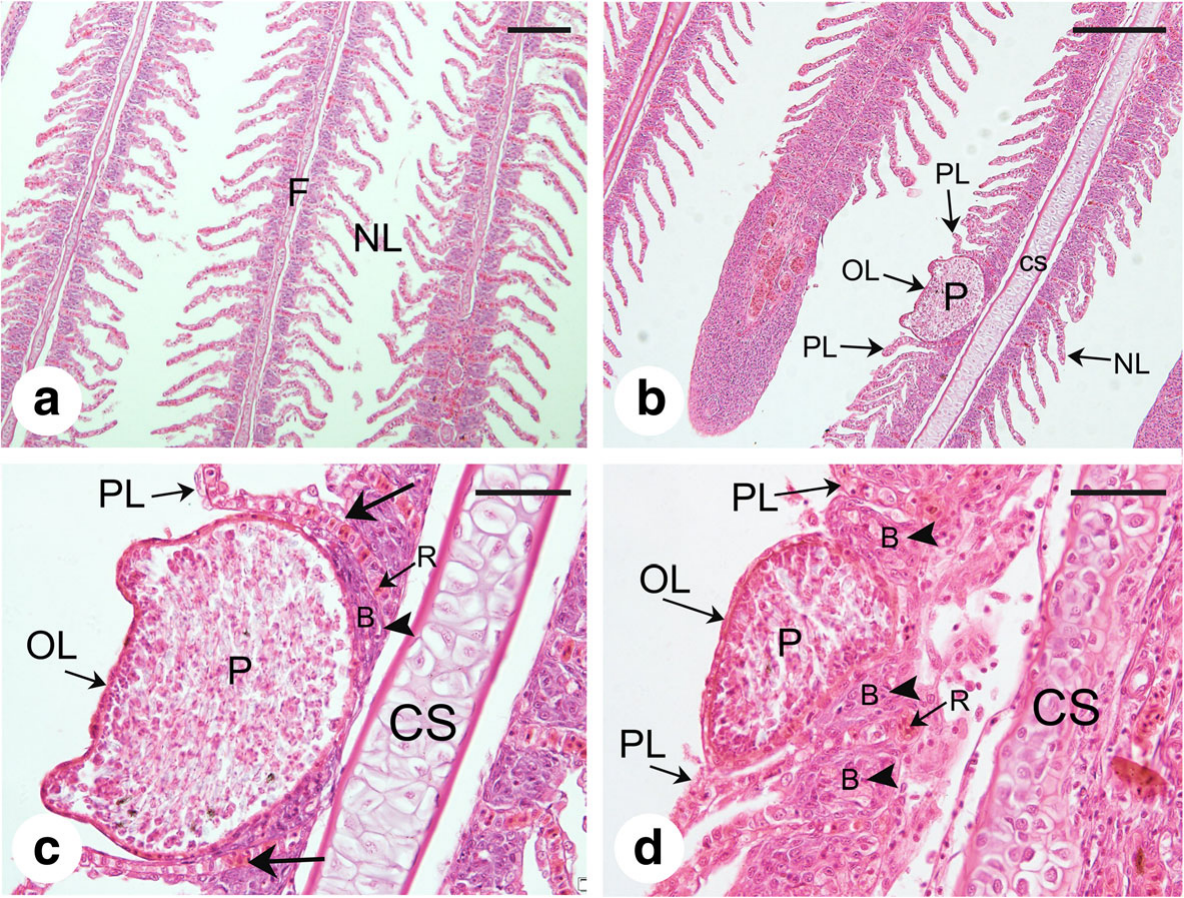
Histological sections of gills of the common carp *Cyprinus carpio* infected by *Myxobolus pseudoacinosus* (narrow type). **a** Normal uninfected gill tissue with parallel filaments and intact gill lamellae. **b** Plasmodia of *M. pseudoacinosus* developing in the lumen of lamellar capillaries. **c, d** Higher magnification showing plasmodia located within the lumen of lamellar capillaries, pushing aside neighbouring lamellae (large arrows) and compressing the undifferentiated basal cells (arrowhead). The cartilaginous structure remains intact. *Abbreviations*: B, undifferentiated basal cells; CS, cartilaginous structure; F, gill filaments; NL, normal gill lamellae; OL, plasmodia-occupied gill lamellae; P, plasmodia; PL, gill lamellae become pushed aside; R, red blood cells. *Scale-bars*: **a**, **b**, 200 μm; **c**, **d**, 50 μm

Histological analysis showed that plasmodia developed in the lumen of lamellar capillaries (Fig. 5b). Compared to the normal gill tissue (Fig. 5a), the gill lamellae neighboring the plasmodia were pushed aside, and the undifferentiated basal cells were compressed (Fig. 5c, d). However, the rest of the gill lamellae and the cartilaginous structure remained intact. No inflammatory responses, including swelling or inflammatory cell infiltrations, were found in any of the histological slides examined.

### Ultrastructure

**Fig. 6:**
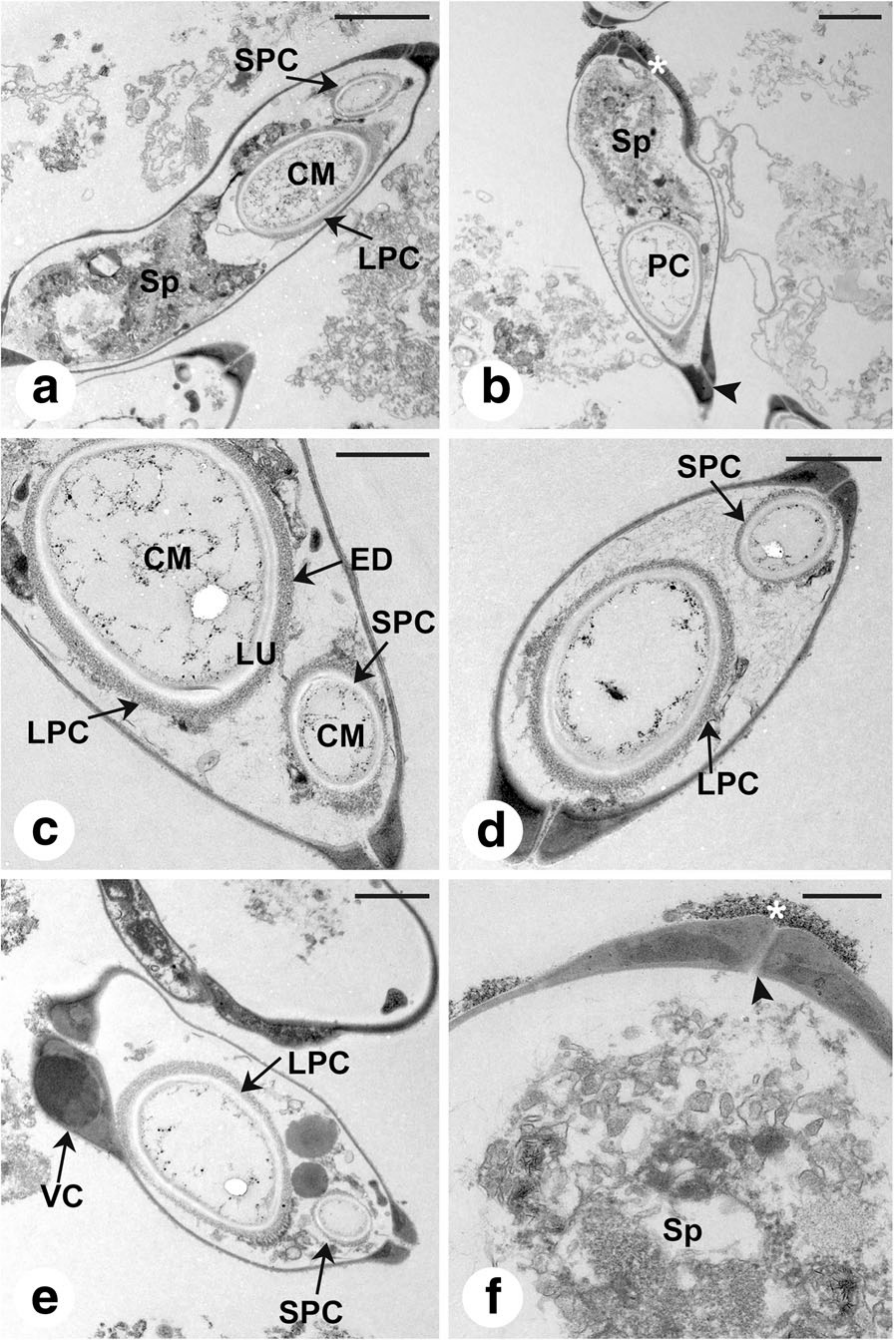
Transmission electron microscopy of *Myxobolus pseudoacinosus* (narrow type) from the gills of *Cyprinus carpio*. **a** Longitudinally sectioned spore showing the large polar capsule, the small polar capsule, the capsule matrix and sporoplasm. **b** Longitudinal section of a spore showing one polar capsule, sporoplasm and the suture line of the valves (arrowhead). Note the electron-dense granules surrounding the posterior end of the spore (white asterisks). **c** Detail of polar capsules showing a three-layer structure consisting of inner capsule matrix, an adjacent electron lucent zone and an outer electron-dense zone. **d** Transversely sectioned spore showing the large and small polar capsule. **e** Immature spore, note the valvogenic cell. **f** Ultrastructural detail of the suture line of the valves (arrowhead) and the electron-dense granules surrounding the posterior end of the spore (white asterisks). *Abbreviations*: CM, capsule matrix; ED, outer electron dense zone; LPC, large polar capsule; LU, adjacent electron lucent zone; PC, polar capsule; SPC, small polar capsule; Sp, sporoplasm; VC, valvogenic cell. *Scale-bars*: **a**, **b**, 2 μm; **c**-**e**, 1 μm: **f**, 500 nm

The presence of a second stunted polar capsule was confirmed by the ultrastructural results (Fig. 6d). Two symmetrical valves, which were joined by a sutural ridge, surrounded the two unequal- sized pyriform polar capsules (Fig. 6a). The polar capsules showed a three-layer structure that consisted of an inner capsule matrix, an adjacent electron-lucent zone and an outer electron-dense zone (Fig. 6c). Numerous sporoplasmosomes and a few vacuoles were observed in the sporoplasm, which was located in the posterior end of myxospore (Fig. 6a, b, f). The distal end of the spore was surrounded by electron-dense granules (Fig. 6b, f). The valvogenic cell could be observed in immature spores (Fig. 6e).

### Molecular analysis

Molecularly, the SSU rDNA sequences obtained from narrow-type and wide-type spores shared high identity (KX586684 *vs* KX810019: 99.6%, identities 1312/1317 bp; KX586684 *vs* KX810020: 99.8%, identities 1042/1044 bp). Blast searches based on the 2025 bases of the SSU rDNA sequence (KX586684) showed almost identical values to *M. paratoyamai* Kato, Kasai, Tomochi, Li & Sato, 2017 (LC228237: 99.3%, identities 1991/2006 bp), and revealed a close relationship between the species of interest and *M. acinosus* (KX810021: 98.6%, identities 1299/1318 bp; KX810022: 98.8%, identities 1933/1957 bp; KX810023: 98.7%, identities 1030/1044 bp), *Myxobolus longisporus* Nie & Li, 1992 (AY36463: 98.7%, identities 2003/2029 bp), followed by *M*. *toyamai* (LC010116: 97.6%, identities 1951/2000 bp; LC010115: 97.5%, identities 1949/2000 bp; HQ338729: 97.2%, identities 1912/1967 bp).

**Fig. 7:**
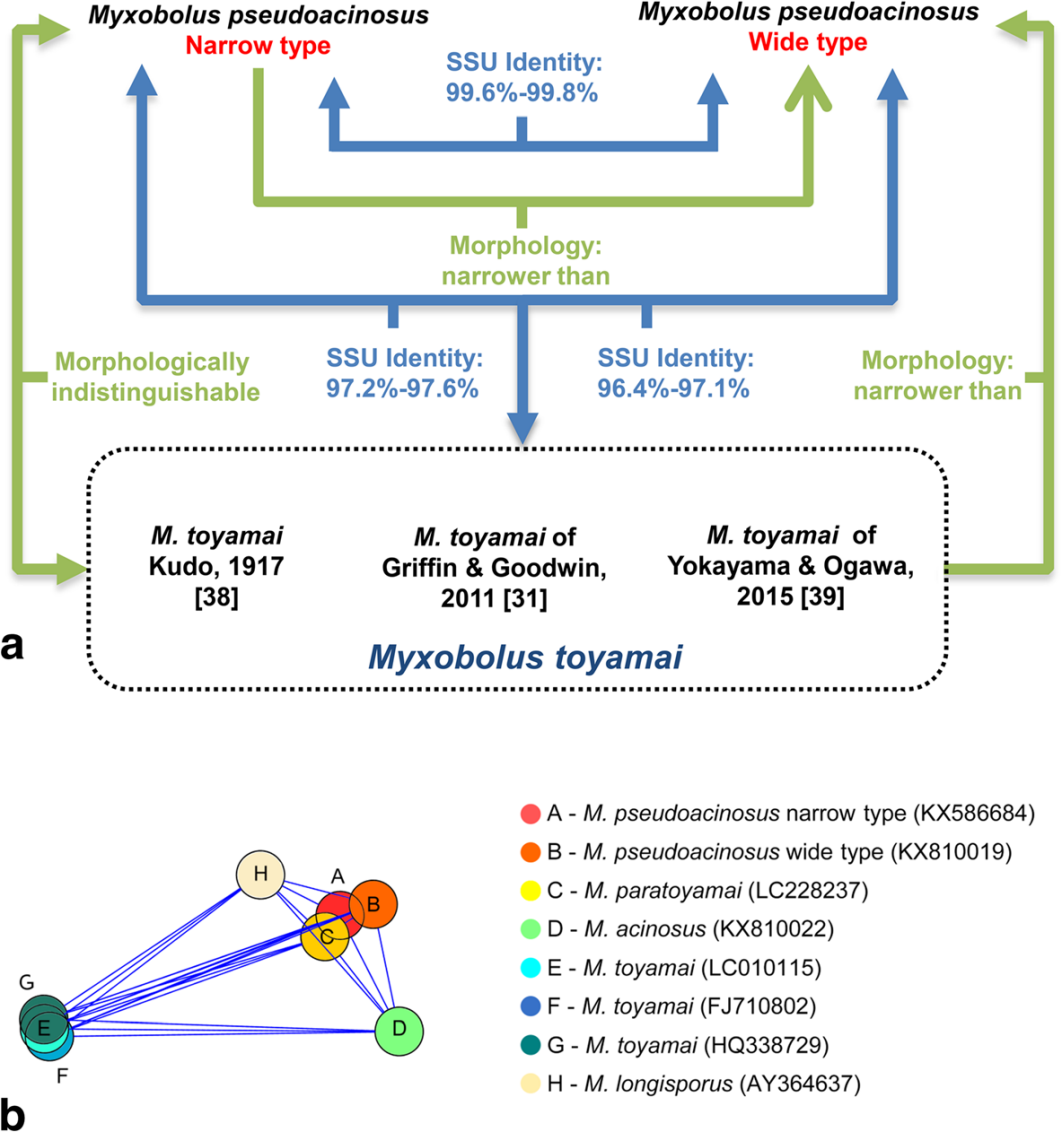
Species validity assessment of *Myxobolus pseudoacinosus*. **a** Schematic diagram showing the general situation of the present study. **b** Visualization of the genetic distance matrix between the SSU rDNA sequence pairs of *M*. *pseudoacinosus* and closely related species (see Table 2 for details)

Results of evolutionary divergence over SSU rDNA sequence pairs between the present species and the closely related *Myxobolus* species are listed in Table 2 and visualized in Fig. 7b. The p-distance values for the present species compared with *M. longisporus* (0.010), *M. acinosus* (0.013), *M. toyamai* (0.033–0.034) are far greater than the intraspecific p-distance of *M. pseudoacinosus* (0.003–0.005) and *M. toyamai* (0–0.001).

**Table 2:** Genetic distances (bellow the diagonal) and sequence similarities (in %, above the diagonal) of *Myxobolus pseudoacinosus* and closely related *Myxobolus* spp. based on SSU rDNA sequence data

	Species (GenBank ID)	1	2	3	4	5	6	7	8	9	10
1	Narrow type (KX586684)		99.6	99.8	99.3	98.6	98.8	97.5	97.4	97.2	98.7
2	Wide type (KX810019)	0.004		99.5	98.9	98.5	98.5	96.4	96.4	96.4	98.2
3	Wide type (KX810020)	0.005	0.009		99.5	98.6	98.8	97.0	97.1	97.1	98.8
4	*M. paratoyamai* (LC228237)	0.003	0.006	0.008		98.3	98.6	97.7	97.7	97.5	98.6
5	*M. acinosus* (KX810021)	0.013	0.016	0.018	0.015		100	96.1	96.2	96.2	97.8
6	*M. acinosus* (KX810022)	0.013	0.016	0.018	0.015	0.000		97.1	97.1	97.1	98.2
7	*M. toyamai* (LC010115)	0.034	0.038	0.039	0.034	0.041	0.041		99.8	99.5	97.4
8	*M. toyamai* (FJ710802)	0.033	0.036	0.038	0.033	0.040	0.040	0.001		99.8	97.3
9	*M. toyamai* (HQ338729)	0.033	0.036	0.038	0.033	0.040	0.040	0.001	0.000		97.1
10	*M. longisporus* (AY364637)	0.010	0.014	0.015	0.010	0.020	0.020	0.031	0.030	0.030

**Fig. 8:**
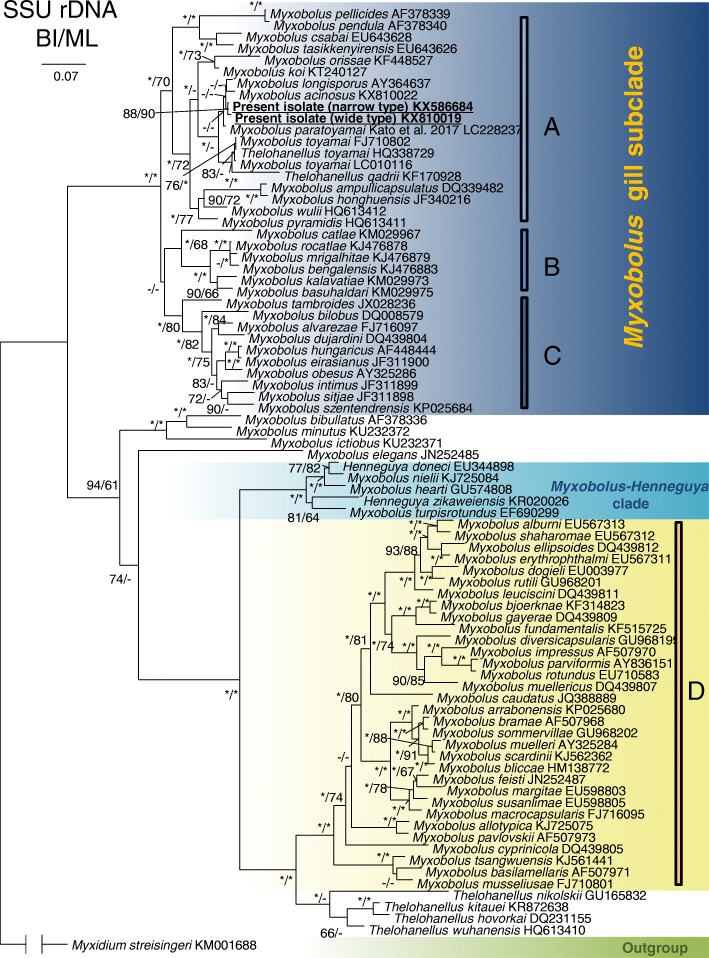
SSU rDNA phylogeny of *Myxobolus pseudoacinosus* and other selected myxozoan species obtained by Bayesian inference analysis. Numbers at branching nodes are Bayesian posterior probabilities/bootstrap values of maximum likelihood analysis (in %). Asterisks represent values ≥ 95%. Dashes represent values ≤ 60% or alternative maximum likelihood topology

Phylogenetic analysis revealed that the present species was positioned within the well-supported *Myxobolus* gill-infecting subclade and clustered with several myxozoans that exclusively parasitize the gill of cyprinid fish: *M. longisporus* (AY36463); *M. acinosus* (KX810022); *M. toyamai* (LC010116, HQ338729, FJ710802); and *Thelohanellus qadrii* (KF170928) (Fig. 8).

## Discussion

To facilitate species identification, the spores collected from the gills of common carp in 2013 and 2015 were studied using a holistic approach comprising spore morphology, biological traits and DNA data. Morphologically, the spores collected in 2013 possessed obviously wider spores and wider large polar capsules than those collected in 2015. Molecular analysis revealed that the two myxozoan samples share almost identical SSU rDNA sequences (99.6–99.8%). Considering the fact that intraspecific morphometric variation [11] and morphotypes [10, 32] are common in myxozoans, the two samples from the gills of common carp were considered to be conspecific with each representing a morphotype of the same species, i.e. wide type (spores collected in 2013) and narrow type (spores collected in 2015).

A holistic comparison between spores of these two morphotypes and the related *Myxobolus* spp. infecting the common carp was also undertaken. The wide-type spores show high similarity with a recently described species *M. paratoyamai* Kato, Kasai, Tomochi, Li & Sato, 2017 [12] in morphology, biological traits and SSU rDNA. Thus, the wide-type morph and *M. paratoyamai* Kato, Kasai, Tomochi, Li & Sato, 2017 were confidently considered to be the same species.

Interestingly, the narrow-type spores showed highly similar morphology to *M*. *toyamai* and molecular comparison revealed 2.4–2.8% differences in SSU rDNA sequences (KX586684 *vs* LC010116: 97.6%; KX586684 *vs* LC010115: 97.5%; KX586684 *vs* HQ338729: 97.2%). According to previous research [33], intraspecific SSU rDNA differences for myxozoans typically range from 0–3.6% and can even be as high as 5% in the case of *Myxobolus pseudodispar* Gorbunova, 1936 [34]. The 2.4–2.8% SSU rDNA differences between the narrow-type spores and *M. toyamai* were thus not enough to designate them as different species. However, based on the classification system of Molnár [35], the infection site of narrow-type spores (intralamellar-vascular, LV3) (Additional file 1: Figure S1) and *M. toyamai* (intrafilamental-epithelial, FE) are different [31]. Despite the totally different tissue-specificity between the narrow-type spores and *M. toyamai*, there have been cases in which one myxozoan species was found to infect multiple tissues/organs [34, 36]. Taken together, these data do not allow narrow-type spores and *M. toyamai* to be easily distinguished and their relationship needs further investigation.

In order to resolve the taxonomic dilemma encountered here, a hypothesis was proposed: narrow-type spores and *M. toyamai* are conspecific. Based on this hypothesis, *M. toyamai* would be designated as a species with intraspecific SSU rDNA divergence of 3.6%. However, this can give rise to new problems in explaining the fact that SSU rDNA sequence of the present species is more similar to that of *M. paratoyamai*, *M. acinosus* (a valid species morphologically distinct from *M. toyamai*, see [2]) and *M. longisporus* (a valid species morphologically distinct from *M. toyamai*, see [37]) than to *M. toyamai* itself (Fig. 7b). Correspondingly, it will also be inappropriate and illogical to put *M. paratoyamai* Kato, Kasai, Tomochi, Li & Sato, 2017, *M. acinosus* and *M. longisporus* along with the present species in the *M. toyamai* “complex” and designate three new morphotypes. Thus, the hypothesis about the conspecificity of the narrow type and *M. toyamai* is not supported by the data presented here.

Based on the data presented above, the species described in this work is herein designated as *M. paratoyamai* Kato, Kasai, Tomochi, Li & Sato, 2017, but with two morphotypes [12]. Considering that this species name was preoccupied by *Myxobolus paratoyamai* Nie & Li, 1992 [2, 30], the replacement name *Myxobolus pseudoacinosus* nom. nov. is proposed. Although lacking molecular data, *M. paratoyamai* Nie & Li, 1992 can be easily distinguished from the present species.

## Conclusions

In conclusion, this study addressed the taxonomic dilemma phenomenon, using *M*. *pseudoacinosus* as a case study. The key considerations to bear in mind when dealing with future taxonomic dilemmas are: (i) intraspecific morphometric variation and morphotypes do exist among myxozoan populations; and (ii) at least for *Myxobolus* species, the co-occurrence of indistinguishable spore morphology and a SSU rDNA difference as low as 2.4% does not necessarily define conspecificity. Moreover, this study is not without limitations. One improvement that can be made is to obtain extra histological and ultrastructural data from wide-type spores, thus making it possible to more thoroughly compare the two morphotypes. Another improvement involves a broader sampling of host specimens, which would enable us to enlarge extensively the view of the myxozoan genetic and morphologic variability. Future work will focus on improving our understanding of the biology of this species and of the variant characteristics of *Myxobolus* species more broadly.

## Additional file

Additional file 1: **Figure S1.** The intralamellar vascular site preference of *M. pseudoacinosus* in gills. **a** The plasmodia of *M. pseudoacinosus* developed in the lumen of lamellar capillaries. The area highlighted in blue represents the gill lamellae. **b** The site preference type of *M. pseudoacinosus* met the definition of intralamellar vascular type 3 (LV3) from the classification system of Molnár [35]. *Abbreviations*: P, plasmodia; LV, intralamellar vascular. (TIF 4655 kb)

## Abbreviations

AIC: Akaike information criterion
FE: Intrafilamental-epithelial
LV3: Intralamellar-vascular
SD: Standard deviation
SSU rDNA: Small subunit ribosomal deoxyribonucleic acid
