# Supplementary figures and images for: Morphological plasticity in Myxobolus Bütschli, 1882: a taxonomic dilemma case and renaming of a parasite species of the common carp
Source: Parasit Vectors. 2018 Jul 9;11:399. doi: 10.1186/s13071-018-2943-0 (PMC6038286; doi:10.1186/s13071-018-2943-0)

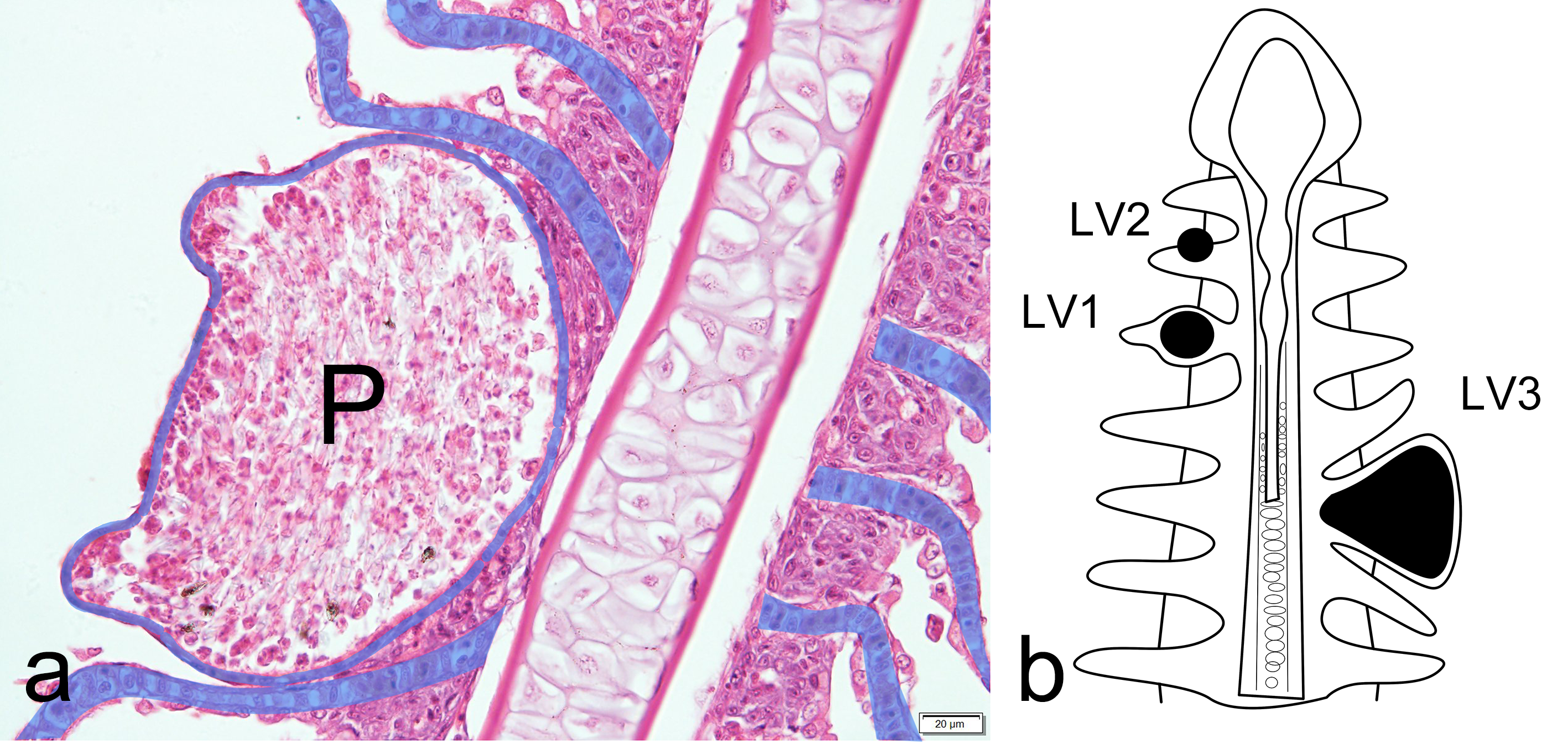

Supplement: Supplementary file 1 — Figure S1. The intralamellar vascular site preference of M. pseudoacinosus in gills. a The plasmodia of M. pseudoacinosus developed in the lumen of lamellar capillaries. The area highlighted in blue represents the gill lamellae. b The site preference type of M. pseudoacinosus met the definition of intralamellar vascular type 3 (LV3) from the classification system of Molnár [35]. Abbreviations: P, plasmodia; LV, intralamellar vascular. (TIF 4655 kb) [file 13071_2018_2943_MOESM1_ESM.tif]
